# Improving the Prognosis of Pancreatic Cancer Through Early Detection: Protocol for a Prospective Observational Study

**DOI:** 10.2196/26898

**Published:** 2021-10-22

**Authors:** Reiko Yamada, Shuji Isaji, Takehiro Fujii, Shugo Mizuno, Masashi Kishiwada, Yasuhiro Murata, Aoi Hayasaki, Hiroyuki Inoue, Yuhei Umeda, Kyosuke Tanaka, Yasuhiko Hamada, Junya Tsuboi, Toshio Kato, Yoshihiro Kondo, Shinsuke Matsuda, Noriko Watanabe, Toru Ogura, Satoshi Tamaru

**Affiliations:** 1 Clinical Research Support Center Mie University Hospital Tsu Japan; 2 Department of Endoscopy Mie University Hospital Tsu Japan; 3 Department of Surgery Toyama Hospital Tsu Japan; 4 Department of Internal Medicine Takeuchi Hospital Tsu Japan; 5 Department of Surgery Nagai Hospital Tsu Japan; 6 Department of Gastroenterology Mie Chuo Medical Center Tsu Japan

**Keywords:** pancreatic cancer, prognosis, early diagnosis, risk factors, scoring system, referral

## Abstract

**Background:**

Pancreatic cancer is associated with high mortality and its rates of detection are very low; as such, the disease is typically diagnosed at an advanced stage. A number of risk factors for pancreatic cancer have been reported and may be used to identify individuals at high risk for the development of this disease.

**Objective:**

The aim of this prospective, observational trial is to evaluate a scoring metric for systematic early detection of pancreatic cancer in Mie Prefecture, Japan.

**Methods:**

Eligible patients aged 20 years and older will be referred from participating clinics in the Tsu City area to the Faculty of Medicine, Gastroenterology, and Hepatology at Mie University Graduate School, until September 30, 2022. Participants will undergo a detailed examination for pancreatic cancer. Data collection will include diagnostic and follow-up imaging data and disease staging information.

**Results:**

The study was initiated in September 2020 and aims to recruit at least 150 patients in a 2-year period. Recruitment of patients is currently still underway. Final data analysis is expected to be complete by March 2025.

**Conclusions:**

This study will provide insights into the feasibility of using a scoring system for the early detection of pancreatic cancer, thus potentially improving the survival outcomes of diagnosed patients.

**Trial Registration:**

UMIN-CTR Clinical Trials Registry UMIN000041624; https://tinyurl.com/94tbbn3s

**International Registered Report Identifier (IRRID):**

DERR1-10.2196/26898

## Introduction

Pancreatic cancer is the fourth most common cause of cancer-related death in Japan, and the number of cases is increasing annually [1]. Prognosis for individuals diagnosed with pancreatic cancer is poor, with a 5-year survival rate of 7.9% reported for patients diagnosed between 2006 and 2009 [2]. Pancreatic ductal adenocarcinoma, referred to simply as pancreatic cancer hereafter, is among the most lethal forms of the disease, with an overall 5-year survival rate of approximately 5% [2]. The overwhelming majority of patients with pancreatic cancer present with locally advanced or distant metastatic disease (80%-85%), and only a minority of patients have surgically resectable tumors [3-5]. Following initial evaluation, only 15%-20% of patients undergo resection [6-8]. In many cases, symptoms manifest only when the cancer reaches an advanced stage, such that by the time it is detected, the cancer may be unresectable. Such late detection is among the main reasons for the poor prognosis of patients with pancreatic cancer.

The Japan Pancreatic Cancer Registry has reported 5-year survival rates of 85.8%, 68.7%, and 59.7% for patients with Stage 0, Ia, and Ib disease, respectively [9]. The 5-year survival rate of patients with pancreatic tumors <10 mm (TS1a) approaches 80.4% and that of patients with Union for International Cancer Control (UICC) Stage 0 is 85.8% [6-10]. Therefore, early detection, which enables multidisciplinary treatment including surgical resection, chemotherapy, and radiation therapy, is key to improving prognosis. However, in Japan, the proportion of cases detected at Stage 0, Ia, and Ib is only 1.7%, 4.1%, and 6.3%, respectively [9], indicating that rates of early detection remain low.

Risk factors for pancreatic cancer include family history, diabetes mellitus, chronic pancreatitis, complication of intraductal papillary mucinous neoplasm, smoking, and excessive alcohol intake [11], but an efficient system to detect patients from high-risk groups has not yet been established. A multicenter retrospective study of early stage pancreatic cancer in Japan indicated a role for screening in individuals without symptoms and demonstrated the importance of imaging modalities including computed tomography (CT), active endoscopic ultrasound (EUS), and magnetic resonance imaging (MRI) for identifying abnormalities warranting further investigation [12]. In a previous study, early detection of pancreatic cancer on a regional basis via cooperation between a regional core hospital and the medical association, using EUS and MRI for diagnostic purposes, was effective for detecting Stage 0 pancreatic cancer [13], and another regional study reported the effectiveness of a similar system [14]. Therefore, we aim to implement a system for the early detection of pancreatic cancer in a regional referral setting in Mie Prefecture, Japan.

We hypothesize that referral of subjects for further examinations based on a scoring metric that assesses the presence of known pancreatic cancer risk factors is a feasible strategy to improve rates of early identification of patients with pancreatic cancer compared with current diagnostic practices.

## Methods

### Study Design and Population

**Figure 1:**
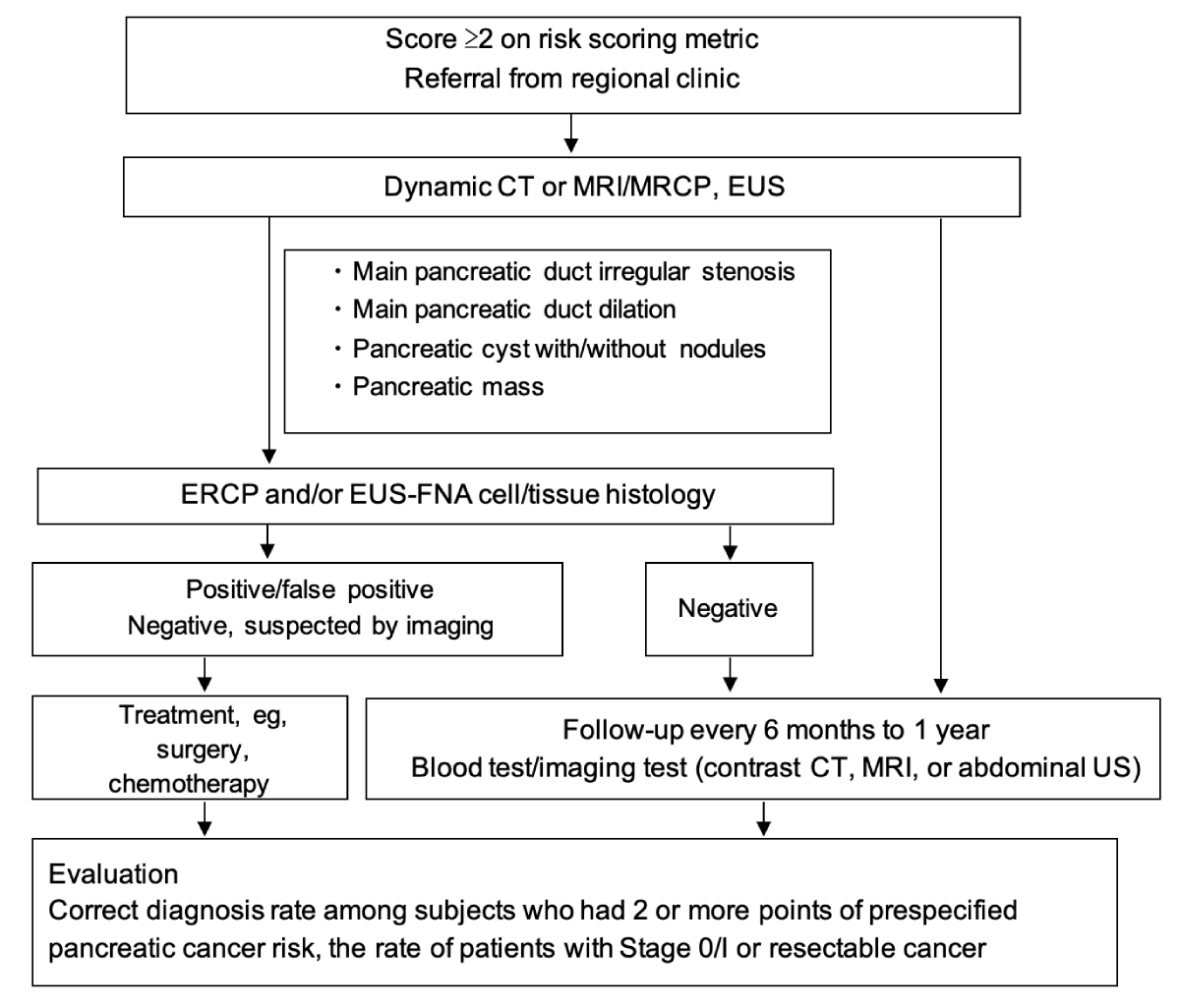
Flowchart of patient enrolment and evaluation. CT: computed tomography; MRI: magnetic resonance imaging; MRCP: magnetic resonance cholangiopancreatography; ERCP: endoscopic retrograde cholangiopancreatography; EUS: endoscopic ultrasound; EUS-FNA: endoscopic ultrasound-guided fine needle aspiration; US: ultrasound.

This is a prospective, multicenter, observational cohort study for the early detection of pancreatic cancer in Japanese patients. The study was initiated in September 2020 and aims to recruit at least 150 patients. The aim of the study is to assess whether using a scoring metric to screen for known pancreatic cancer risk factors will improve the rate of early identification in this patient population. The flowchart of patient enrolment and evaluation is summarized in Figure 1. The study is registered in UMIN-CTR Clinical Trials Registry (UMIN000041624). Patients who seek consultation at one of five participating local clinics in the Tsu City area until September 30, 2022, will be referred through the regional medical reference system to the Faculty of Medicine, Gastroenterology, and Hepatology at Mie University Graduate School for a detailed examination for pancreatic cancer. 

Prespecified risk factors and associated point scores will be assessed as follows: symptoms (abdominal pain, jaundice, back pain, or weight loss): 1 point; newly diagnosed diabetes mellitus (type 2) or worsening of diabetes: 1 point; family history of pancreatic cancer (parent, child, or siblings, including other types of pancreatic neoplasms): 1 point; abnormal serum amylase level (under 44 U/mL or over 132 U/mL): 1 point; elevated serum CA19-9 level (>37 U/mL [15]): 1 point; pancreatic duct dilation (>3 mm) or pancreatic cyst detected by abdominal ultrasound: 2 points [11]. Adults aged 20 years or older scoring ≥2 points on this metric for prespecified pancreatic cancer risk factors will be eligible for inclusion (those scoring 2 or less but at high risk for pancreatic cancer will be referred for follow-up, but not included in the study). The total number of patients diagnosed with pancreatic cancer and the subsets of patients with surgically resectable pancreatic cancer and Stage 0/I pancreatic cancer will be determined (Figure 1). After registration, participants will undergo hospital visits every 6 months for a period of 2 years.

### Diagnostic Procedures

Diagnostic data will be collected from patients referred to Mie University Graduate School of Medicine and will include the outcomes of routine EUS, dynamic CT, and MRI or magnetic resonance cholangiopancreatography assessments. Confirmatory diagnostic tests will include endoscopic retrograde cholangiopancreatography and endoscopic ultrasound-guided fine needle aspiration. Biopsy samples collected during EUS-FNAB will be fixed in alcohol and stained using the Papanicolaou multichromatic procedure. The remaining material will be fixed in 10% formalin and embedded in paraffin for cell block analysis to obtain histological diagnosis using hematoxylin and eosin staining. Tumor staging will be performed according to the UICC-American Joint Committee on Cancer tumor, node, and metastasis categories [16].

### Follow-Up Assessments

Patients with a diagnosis of pancreatic cancer will undergo appropriate treatment (eg, surgery and/or chemotherapy) according to the standard of care guidelines [11]. Patients with negative diagnostic results will be followed by contract CT, abdominal ultrasound, and/or MRI. Blood testing for carcinoembryonic antigen (CEA), carbohydrate antigen 19-9 (CA19-9), amylase, and lipase will be performed at intervals of 6 months.

### Study Endpoints

The primary endpoint is the diagnosis rate (number of patients diagnosed/total number of study participants) for pancreatic cancer, calculated with a 95% CI. The secondary endpoints include the diagnosis rates of pancreatic cancer by stage, pancreatic neoplasms other than pancreatic ductal adenocarcinoma, Stage 0/I pancreatic cancer at each visit, and surgical resection. For patients with a definite diagnosis of pancreatic cancer, a frequency table will be created based on the resectability classification [16].

### Sample Size

We based our sample size on data from a previous study, in which 28 patients were diagnosed with pancreatic cancer among 224 subjects who had 2 or more pancreatic cancer risks [14]. The risks prespecified in this study are considered equivalent to those reported previously, meaning that the rate of the patients diagnosed with pancreatic cancer is expected to be approximately 10%. To obtain estimation accuracy (95% CI) of ±5%, the required number of subjects is calculated as 138. The planned number of participants in this study is 150. We expect that approximately 100-150 patients with ≥2 points on the pancreatic cancer risk scoring metric will visit the participating clinics per year and that 80% of these eligible patients will give consent to participate in the study [14]. Therefore, in the 2-year referral period, 160-240 participants are expected to enroll in this study. However, when the number of patients reaches 150, registration will continue because this a prospective observational study without any intervention.

### Statistical Analyses

Patient characteristics will be summarized using descriptive statistics with frequency, mean, and standard deviation. The following items will be evaluated: sex, age, symptoms suggestive of pancreatic cancer (abdominal pain, jaundice, back pain, and weight loss), newly diagnosed or worsening of type 2 diabetes, family history of pancreatic cancer, abnormal laboratory test value for serum amylase (high or low) or serum CA19-9 (high), pancreatic duct dilation, or pancreatic cyst detected by echography. Participants’ BMI and smoking status will also be recorded for use in adjusted analyses.

Loss of participants to follow-up will be minimized by monitoring missed appointments and contacting participants promptly. Where key data items are unavailable or only partially reported, we will use multiple imputation to impute missing data where indicated.

Data on patients with a confirmed diagnosis will be summarized using descriptive statistics according to resectability classification [16]. Binomial logistic regression analysis (both univariate and multivariate analyses) will be performed with a diagnosis of pancreatic cancer as the objective variable and each risk factor and the result of an imaging test as explanatory variables. For the primary multivariable analysis, variables will be selected with a two-sided significance level of 5%. In addition to the unadjusted analyses, logistic regression analyses adjusted for BMI and smoking status will be conducted.

Sensitivity analysis will be conducted to generate multivariable prediction scores based on all available risk factors, regardless of performance or significance on univariable analysis, and based on alternate combinations of risk factors as indicated. Formal comparisons of the relative goodness-of-fit of each model will be conducted through derivation of the Akaike Information Criteria and/or the Bayesian Information Criteria as indicated. We will compare the performance of the prediction score based on this primary model against those of the sensitivity analysis models.

No interim analysis or subgroup analysis will be performed. A separate follow-up study will be conducted to evaluate the 5-year survival rate of pancreatic cancer in the region. SPSS statistical software (Version 25.0, IBM Corp) will be used for all analyses.

### Ethical Approval and Informed Consent

This study protocol has been approved by the ethics committee of Mie University Graduate School of Medicine (approval number H2020-143). All participants will provide written informed consent prior to study enrolment.

## Results

The study was initiated in September 2020 and aims to recruit at least 150 patients in a 2-year period. Recruitment of patients is currently still underway. Final data analysis is expected to be complete by March 2025.

## Discussion

The detection of pancreatic cancer, particularly at the early stages when 5-year survival rates are relatively high, remains challenging. Despite advancement in the knowledge of potential risk factors that cause pancreatic cancer and newly available tools for early diagnosis, its incidence is increasing and is estimated to include 355,317 new cases per year up to 2040 [17]. Previous studies have revealed the value of using clinical findings to detect early pancreatic cancer [12-14]. Sakamoto et al [14] found that of the five clinical findings they investigated, three (symptoms, new onset diabetes, and high CA19-9) were more frequent in the advanced pancreatic cancer group than in the early and non–pancreatic cancer groups. In contrast, high amylase and/or high pancreatic amylase levels were significantly more frequent in the early pancreatic cancer group than in the other groups. Similarly, CA19-9 levels >37 U/mL had a sensitivity, with 95% specificity, of 68% and 53% for detecting pancreatic cancer at >1 year and >2 years before diagnosis, respectively [18].

We plan to initiate a prospective, observational study designed to evaluate a scoring metric for systematic early detection of pancreatic cancer in Mie Prefecture, Japan. If demonstrated to be effective, our system may represent a novel tool for use in the diagnosis and subsequent management of this patient population. A strength of our study is that, although patients will be referred from five different participating centers, all diagnostic and follow-up assessments will be performed at a single institution, eliminating variability in local practices. A possible limitation is that, for ethical reasons, this study will not directly compare the scoring metric with no scoring in terms of new diagnoses. However, the rates of diagnoses, particularly of early stage pancreatic cancer, can be compared with historic published data. Additionally, with a relatively small sample size, this initial study will be limited in its ability to be applied to the wider population and different ethnicities because most of the participants are likely to be Japanese. A planned future study will examine 5-year survival after referral using the scoring metric and the value of each item in the scoring metric for detecting early stage pancreatic cancer in a larger number of patients.

## Abbreviations

CA19-9: carbohydrate antigen 19-9
CEA: carcinoembryonic antigen
CT: computed tomography
EUS: endoscopic ultrasound
MRI: magnetic resonance imaging
UICC: Union for International Cancer Control
